# Vaccine-Induced Immunity in Children and Adolescents After Chemotherapy for Acute Lymphoblastic Leukemia: A Systematic Review

**DOI:** 10.3390/vaccines14050419

**Published:** 2026-05-07

**Authors:** Cláudia F. C. Valente, Heloisa Ihle Garcia Giamberardino, Tânia Cristina de Mattos Barros Petraglia, Cristiane Feitosa Salviano, Priscilla Lemos Gomes, Roberia Mendonça de Pontes, Maria Eduarda Canellas de Castro, Lis R. V. Antonelli, Olindo Assis Martins-Filho, Cleandro Pires de Albuquerque, Licia Maria Henrique da Mota

**Affiliations:** 1 Hospital da Criança de Brasília José Alencar, Brasília 70684-831, Brazil; crisenf.salviano@gmail.com (C.F.S.); roberia.pontes@hcb.org.br (R.M.d.P.); 2 Hospital Pequeno Príncipe, Curitiba 80250-060, Brazil; heloisa.ihle@gmail.com; 3 Saúde da Criança e do Adolescente, Faculdade de Medicina Estácio de Sá, Rio de Janeiro 20071-004, Brazil; taniapetraglia@gmail.com; 4 Hospital Materno-Infantil de Brasília, Brasília 70684-831, Brazil; priscillalegos@gmail.com; 5 Faculdade de Medicina, Universidade de Brasília, Brasília 70684-831, Brazil; maria.castro@unb.br; 6 Instituto René Rachou, Fundação Oswaldo Cruz—FIOCRUZ-Minas, Belo Horizonte 30190-003, Brazil; lis.antonelli@fiocruz.br (L.R.V.A.); oamfilho@gmail.com (O.A.M.-F.); 7 Programa de Pós-Graduação em Ciências Médicas, Universidade de Brasília, Brasília 70684-831, Brazil; cleandro.albuquerque@gmail.com (C.P.d.A.); liciamhmota@gmail.com (L.M.H.d.M.)

**Keywords:** acute lymphoblastic leukemia, chemotherapy, vaccine, immunosuppression

## Abstract

Background: Acute lymphoblastic leukemia is the most prevalent childhood cancer and the leading cause of cancer mortality before the age of 20. Although therapeutic advances have significantly improved survival, children and adolescents treated for acute lymphoblastic leukemia remain vulnerable to infections, largely preventable by vaccination, due to humoral and cellular immune dysfunction induced by disease and treatment. Materials and Methods: This systematic review, based on electronic databases, aims to evaluate antibody levels associated with potential protective immunity against vaccine antigens for diphtheria, pertussis, tetanus, poliomyelitis, *Haemophilus influenzae* type b, measles, mumps, rubella, influenza, varicella-zoster virus, yellow fever, pneumococcal, and meningococcal diseases in children and adolescents treated for acute lymphoblastic leukemia after completion of chemotherapy. Results: A total of twenty-four studies published between 1981 and 2023 were included, comprising 1110 children and adolescents. Protective antibody levels ranged from 11% to 97% for diphtheria, 0% to 90% for pertussis, 20% to 100% for tetanus, and 11% to 95% for poliomyelitis. *Haemophilus influenzae* type b, protection ranged from 16.7% to 100%. Viral vaccines also showed heterogeneous responses, with protection rates of 25–79% for mumps, 16–86% for measles, 35–98% for rubella, and 23–75% for varicella-zoster virus. Antibody responses to pneumococcal and meningococcal vaccines were consistently low, with protection rates of 5–38% for pneumococcal studies and 12% in a single meningococcal study. Conclusions: This review found a consistent and clinically relevant loss of vaccine-induced immunity in children and adolescents treated for acute lymphoblastic leukemia. The recommendation of vaccine booster doses for this vulnerable population, irrespective of serological status, may represent a more practical approach to ensuring adequate post-chemotherapy treatment protection.

## 1. Introduction

Acute lymphoblastic leukemia (ALL) is the most common cancer among children and the most frequent cause of death from cancer before 20 years of age [1]. Advances in the optimal use of antileukemic agents, improved supportive care, and refined risk stratification have markedly increased the 5-year event-free survival rates over recent decades, from approximately 60% to nearly 90%. These clinical improvements have been accompanied by a deeper understanding of ALL pathobiology, mechanisms of drug resistance, and the pharmacokinetics of antileukemic therapies in the host [2].

Despite these therapeutic successes, children and adolescents with hematological malignancies such as ALL remain at high risk of infections due to combined humoral and cell-mediated immune dysfunction. A substantial proportion of these infections is caused by vaccine-preventable diseases (VPDs). However, vaccination in this population is frequently overlooked in routine oncologic care, often due to limited training among hematologists or concerns regarding safety, optimal timing, and the selection of vaccines to initiate. Moreover, there is currently considerable variability in vaccine acceptance and practices, even among healthcare professionals [3].

Intensive chemotherapy, while essential for achieving high rates of cure, results in significant impairment of immunocompetence by the end of treatment. Both humoral and cellular immune compartments are affected, and immune recovery typically requires 6 to 12 months. The extent and pace of immune reconstitution may vary according to treatment intensity. Although recovery of transitional B cells and naïve B and T lymphocytes generally occur within months, reconstitution of memory B and T cells may remain incomplete for up to five years, with potential consequences for long-term vaccine-induced immunity and immune responses to revaccination. Regardless of increasing recognition of post-chemotherapy immune impairment, there is still no consensus on whether routine serological testing or universal revaccination should be recommended for children treated for ALL [4,5,6].

The Global Initiative for Childhood Cancer (GICC) of the World Health Organization (WHO), has been working with Ministries of Health to increase the prioritization of childhood cancer treatment at the governmental level, including national policies and monitoring tools to ensure immunizations for this population. After therapy cessation, these children return to their communities and school environments, where effective protection against communicable diseases is essential. Nevertheless, suboptimal immunity frequently leaves them vulnerable to infections. Moreover, the need for additional vaccine doses or revaccination schedules for childhood cancer survivors, including their funding, have not yet been systematically addressed in health policy discussions [7].

This systematic review aims to evaluate antibody levels associated with potential protective immunity against vaccine antigens for diphtheria, pertussis, tetanus, poliomyelitis, *Haemophilus influenzae type b* (Hib), measles, mumps, rubella, influenza, varicella-zoster virus, yellow fever, pneumococcal, and meningococcal diseases in children and adolescents treated for ALL after completion of chemotherapy, with the hypothesis that vaccine-induced immune responses may be impaired in this population.

## 2. Materials and Methods

### 2.1. Overall Description

We conducted a systematic literature review to address the research question: What is the immunization status or protection against VPDs in children and adolescents following chemotherapy for ALL?

The review protocol was registered on the International Prospective Register of Systematic Reviews (Prospero ID number CRD42022382698) and the present reporting followed the Preferred Reporting Items for Systematic Reviews and Meta-Analyses (PRISMA) statement [8].

### 2.2. Search Strategies and Eligibility Criteria

We searched the electronic databases MEDLINE/PubMed, Scopus, EMBASE and Cochrane Library in December 2025. The search strategy was developed using a combination of controlled vocabulary and free-text terms adapted to each database. Medical Subject Headings (MeSH), Emtree terms, and DeCS descriptors were applied whenever available, combined with synonymous keywords related to acute lymphoblastic leukemia, chemotherapy, vaccination, antibody persistence, and seroprotection We combined used descriptors through Boolean operators according to the search strategy, as shown in Supplementary Table S1.

Eligibility criteria included children and adolescents aged 0–18 years who had been treated for ALL, with chemotherapy completed 6–12 months prior to assessment. Participants had not undergone hematopoietic stem cell transplantation (HSCT), were not receiving other immunosuppressive therapies, and had no diagnosis of primary or secondary immunodeficiency.

### 2.3. Record Selection

The literature search results were uploaded to Rayyan of Qatar Computing Research Institute [9], and duplicate articles were removed. Two reviewers independently screened the titles/abstracts of identified references, and a third mediated conflicts. After this phase, full-text screening was performed in the same way.

### 2.4. Data Extraction

Data extraction was performed independently by three reviewers using a standardized data extraction table developed specifically for this review. Extracted variables included study identification (author and year of publication), study design, country, and population characteristics such as mean age of participants, proportion of male participants, type of ALL, intensity of chemotherapy (classified as standard-, intermediate-, or high-risk), mean time of post-chemotherapy evaluation, and reported comorbidities.

In addition, data related to immunization status or immunological protection against vaccine-preventable diseases were extracted. These outcomes included protection or serological response to diphtheria, pertussis, tetanus, poliovirus, Hib, mumps, measles, rubella, influenza, varicella, yellow fever, pneumococcal disease, and meningococcal disease.

### 2.5. Risk of Bias Assessment

The methodological quality and risk of bias of the included studies were assessed using the tool proposed by Munn et al. for systematic reviews of observational epidemiological studies [10]. This instrument evaluates key domains related to study design, participant selection, outcome assessment, and data analysis [10]. Two reviewers independently assessed each study, and any discrepancies were resolved through discussion and consensus. The results of the quality assessment were considered in the interpretation of findings and synthesis of the evidence.

## 3. Results

### 3.1. Study Selection

**Figure 1:**
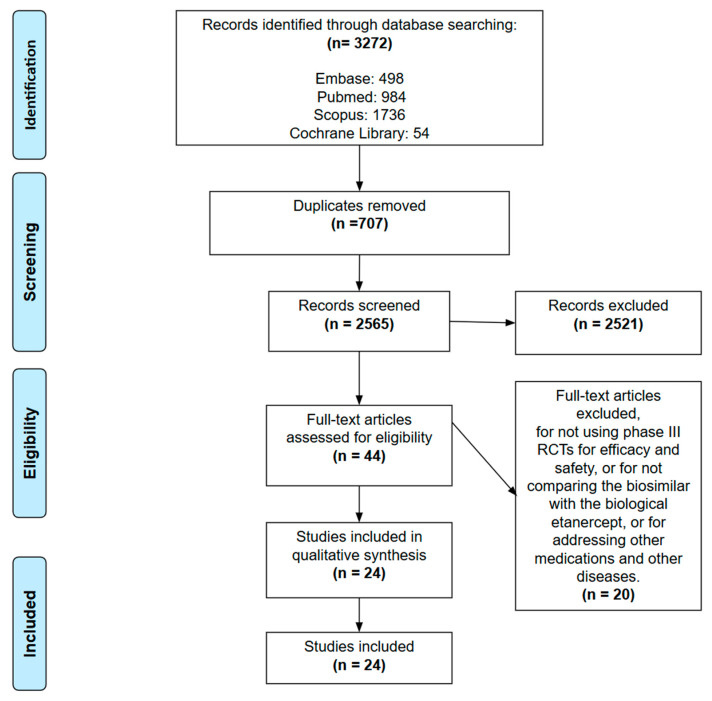
Prisma flow diagram.

The results of our database search in Embase, PubMed, Scopus and Cochrane Library identified 3272 studies. It was found that 707 were duplicate; at first analysis of titles and abstracts, 2521 studies were excluded by the reviewers. After full text analysis, 20 other studies were excluded, resulting in 24 studies selected (Figure 1).

### 3.2. Study Characteristics

A total of twenty-four studies published between 1981 and 2023 were included, comprising 1110 children and adolescents, with a mean age ranging from 3.8 to 10 years, male sex representing 37–69%, and sample sizes varying from 22 to 162 participants from high and middle-income countries (Table 1).

Among the 24 included studies, protective antibody levels ranged from 11% to 97% for diphtheria (8 studies), 0% to 90% for pertussis (4 studies), 20% to 100% for tetanus (12 studies), and 11% to 95% for poliomyelitis (7 studies). For Hib, protection ranged from 16.7% to 100% (5 studies). Viral vaccines also showed heterogeneous responses, with protection rates of 25–79% for mumps (9 studies), 16–86% for measles (15 studies), 35–98% for rubella (10 studies), and 23–75% for varicella-zoster virus. Antibody responses to pneumococcal and meningococcal vaccines were consistently low, with protection rates of 5–38% in two pneumococcal studies and 12% in a single meningococcal study [4,11,12,13,14,15,16,17,18,19,20,21,22,23,24,25,26,27,28,29,30,31,32,33].

**Table 1:** Characteristics of included studies and participants.

Authors	Year	Study Type	Study Country	Sample Size	Male	Age	ALL	Vaccines
Speckhart [32]	2023	Cross-sectional	United States of America	55	27	10	NR	Measles
					50%			Mumps
								Rubella
Anafy [33]	2023	Cross-sectional	Israel	96		6.2		Pertussis
					50		79 SR	Polio
					52%		17 HR	Measles
								Varicella
Pearson [31]	2023	Cross-sectional	United States of America	28		5.5	NR	Tetanus
					14			Measles
					50%			Mumps
								Rubella
								Varicella
Dorval [30]	2021	Cross-sectional	Canada	71	35	3.8	49 SR	Pneumo
					49%		20 IR	
							2 HR	
Toret [29]	2021	Cross-sectional	Turkey	46		6.1	13 SR	Measles
					17		29 IR	Mumps
					37%		4 HR	Rubella
								Varicella
Top [28]	2020	Cohort	Canada	74		8.1	NR	Tetanus
					37			Pertussis
					50%			Pneumo
								Varicella
Fouda [27]	2018	Cross-sectional	Saudi Arabia	57	28	5.3	NR	Measles
					49%			Mumps
								Rubella
Keskin Yildirim and Buyukavci [26]	2018	Cross-sectional	Turkey	162	NR	5.7	NR	Mumps
								Rubella
De La Fuente Garcia [25]	2017	Cross-sectional	Canada	60		4.1		Diphtheria
								Tetanus
					34		37 SR	Polio
					57%		23 HR	Hib
								Measles
								Mumps
								Rubella
Onoratelli [24]	2016	Cross-sectional	Argentina	23	16	9.2	12 SR	Measles
					69%			Mumps
								Rubella
								Varicella
Januszkiewicz-Lewandowska [23]	2015	Case–Control	Poland	40	15	5.1	NR	Diphtheria
					65%			Tetanus
Patel [22]	2014	Cohort	United Kingdom	26	NR	7.9	NR	Varicella
Viana [21]	2012	Case–Control	Brazil	33	NR	10.3	17 SR	Measles
							16 HR	Mumps
								Rubella
Lehrnbecher [20]	2011	Randomized Clinical Trial	Germany	24		7.3	24 SR	Diphtheria
					14			Tetanus
					58%			Poliomyelitis
								Hib
Zengin and Sarper [18]	2009	Case–Control	Turkey	61		10.5	NR	Diphtheria
					12			Tetanus
					60%			Measles
								Hib
Cipolloti [19]	2009	Cross-sectional	Brazil	23		9.2	12 SR	Measles
					16			Mumps
					69%			Rubella
								Varicella
Kosmidis [17]	2008	Cohort	Greece	72		4	26 SR	Polio
					48		42 IR	Measles
					66%		4 HR	Mumps
								Rubella
Patel [15]	2007	Cohort	United Kingdom	59		8	59 SR	Diphtheria
								Tetanus
								Pertussis
					34			Polio
					57%			Hib
								Measles
								Mumps
								Rubella
								Meningo
Calaminus [16]	2007	Cohort	Germany	59	33	5	31 SR	Diphtheria
					56%		28 HR	Tetanus
Volc [14]	2006	Cross-sectional	Brazil	22	NR	8	10 SR	Measles
							12 HR	Rubella
Ercan [13]	2005	Case–Control	Turkey	37		8.4	NR	Diphtheria
					22			Tetanus
					61%			Pertussis
								Measles
								Mumps
Ek [4]	2004	Case–Control	Sweden	31	NR	9.9	6 SR	Diphtheria
							16 IR	Tetanus
							9 HR	Hib
Nilsson [12]	2000	Cohort	Sweden	43	NR	7	NR	Measles
								Rubella
van der Does-van den Berg [11]	1981	Case–Control	The Netherlands	49		6	NR	Diphtheria
					30			Pertussis
					61%			Tetanus
								Polio

Abbreviations: ALL: Acute lymphoblastic leukemia; Hib: *Haemophilus influenzae* type b; Polio: Poliomyelitis; Pneumo: Pneumococcal; Meningo: Meningococcus; NR: Not reported; HR: high risk; IR: intermediate risk and SR: standard risk.

The most frequently evaluated vaccines were measles and tetanus. Measles is considered by the World Health Organization (WHO) an indicator of health system quality, while tetanus is a well-known, highly immunogenic antigen. We present the results for tetanus, measles, and Hib protein, viral, and polysaccharide antigens in Table 2.

No study evaluating influenza or yellow fever met the selection criteria of our systematic review.

The oldest study included in this systematic review was published by van der Berg et al. in 1981 [11]. The authors evaluated antibody titers against protein vaccines in previously vaccinated children with ALL in remission after cessation of therapy and compared them with healthy controls. The study demonstrated lower antibody levels in patients with leukemia compared with healthy children [11].

Assessing live viral vaccines, Nilsson et al. (2000) and Volc et al. reported reduced seroprotection against measles and rubella in children treated for ALL after chemotherapy, with a substantial proportion remaining susceptible, particularly for measles, with seropositivity ranging from 53% to 65% [12,14].

Patel et al. (2007), from the United Kingdom, conducted a study including 59 children aged 1–18 years who had completed standard chemotherapy for acute leukemia. Antibody levels against Hib, tetanus, diphtheria, acellular pertussis, meningococcal C, poliovirus, measles, mumps, and rubella were measured six months after completion of treatment. Protective antibody levels were observed in 100% of patients for tetanus, 87% for Hib, 71% for measles, 12% for meningococcal C, and 11% for all three poliovirus serotypes [15]. The authors highlighted that improved survival rates in children with leukemia are a result of advances in treatment and supportive care; however, both the disease and its therapy led to immunosuppression. Therefore, evaluating the long-term impact of chemotherapy on immunity to vaccine-preventable diseases is essential [15].

Garcia et al. (2017), in a Canadian cohort, evaluated 61 children after chemotherapy and found that fewer than 50% were seroprotected against tetanus, diphtheria, poliovirus type 3, Hib, and mumps [25]. Additionally, no more than 80% were seroprotected against poliovirus types 1 and 2, measles, rubella, and varicella [25].

Onoratelli et al. (2016) assessed humoral immunity to tetanus, measles, and rubella in children with ALL treated in Argentina between 2008 and 2013. Protective antibody levels in the high-risk group were observed in 33% for tetanus, 50% for rubella, and none of the patients for measles [24]. Patients with high-risk ALL had lower protective antibody levels, although one of the limitations of these results is that the high-risk group included only six patients. These findings reinforce the need to reconsider revaccination strategies for patients undergoing chemotherapy for ALL, including those classified as standard, intermediate, or high-risk [24].

Notably, antibody responses to polysaccharide-conjugated vaccines were consistently lower than those observed for protein or live-attenuated vaccines. Dorval et al. (2021) evaluated immunity to *Streptococcus pneumoniae* in children after completion of ALL treatment [30]. All participants had been fully immunized with three doses of pneumococcal conjugate vaccine (PCV) before leukemia diagnosis. At the end of chemotherapy, only 25.6% retained pneumococcal seroprotection. Children treated for ALL were therefore less likely to be protected against invasive pneumococcal disease compared with the general population, and recommended a booster dose after the completion of chemotherapy to confer high rates of seroprotection against invasive pneumococcal disease among children treated for ALL [30].

More recently, in 2023, Anafy et al. published results for ninety-six children (aged 1–18 years at diagnosis) who completed chemotherapy for ALL and had measured antibody levels in the patient’s serum against measles, varicella, polio, and pertussis. The highest percentage of positive serology was against polio (87%) and the lowest against pertussis (4%) (*p* < 0.001), and related differences between patients with high risk (HR) and non-HR ALL regarding serology status for some vaccines (measles, hepatitis A and B) [33]. Speckhart et al. found seroprotection against measles in 64% of 24 ALL children cancer survivors who had received two doses of MMR before cancer diagnosis [32].

### 3.3. Risk of Bias

The methodological quality of the included studies was assessed using the Joanna Briggs Institute (JBI) critical appraisal checklist, as detailed in the Supplementary Materials (Table S2). Overall, most studies were rated as having satisfactory quality, as illustrated by the predominance of positive (“green”) assessments in Table S2. However, two studies presented methodological limitations due to insufficient description of the assays used to measure antibody concentrations against VPDs, and four studies did not clearly define the antibody thresholds used to determine seroprotection. These limitations were considered but did not preclude inclusion of the studies in the review.

## 4. Discussion

While vaccination recommendations for patients undergoing hematopoietic stem cell transplantation are well established, vaccination practices following conventional chemotherapy for ALL remain controversial [3,34].

Vaccines induce protection against VPDs through both humoral and cellular immune responses. Protective immunity relies on the generation of antigen-specific antibodies with adequate specificity, avidity, and neutralizing capacity. Assessment of cellular immune responses is not feasible in routine clinical practice for guiding immunization strategies in pediatric oncology patients. Consequently, humoral immunity assessed by vaccine-specific antibody levels remains the most practical and widely used surrogate marker of vaccine-induced protection [35].

In this systematic review, we evaluated the immunization status and protection against VPDs in children and adolescents after completion of chemotherapy for ALL. Specifically, we assessed serological immunity to vaccines administered before cancer diagnosis, including measles, mumps, rubella, varicella, influenza, diphtheria, tetanus, pertussis, Hib, yellow fever, poliomyelitis, pneumococcal, and meningococcal vaccines. We aimed to generate evidence to support future immunization recommendations for patients following chemotherapy.

A major advance in the treatment of ALL was the introduction, in the 1970s, of an eight-drug induction regimen administered over eight weeks, followed by a consolidation phase. This approach became the foundation of the Berlin–Frankfurt–Münster (BFM) protocol, which underpins most contemporary ALL therapies worldwide and has contributed to survival rates exceeding 90% in high-income countries. The remarkable improvement in survival over the past five decades has not resulted from the development of new drugs, but rather from the optimization of dosing and treatment strategies based on risk stratification. This classification determines treatment intensity and, consequently, the degree of immunosuppression, which may affect the immune response to vaccines.

The studies included in this review span more than four decades, a period during which both vaccination schedules and laboratory assays have undergone substantial changes. Differences in national immunization programs, including the introduction of new vaccine formulations (e.g., conjugate vaccines) and modifications in dosing schedules, may have influenced baseline immunity across study populations. In addition, advances in serological methods—from earlier assays with limited sensitivity and specificity to more standardized and quantitative techniques—may have affected the measurement and comparability of antibody levels over time. These factors likely contribute to the heterogeneity observed across studies and should be considered when interpreting the findings.

We included 24 studies conducted across continents in multiple represented countries with different income levels. All studies demonstrated a negative impact of chemotherapy on vaccine-induced immunity, with reduced seroprotection observed after treatment, and recommended booster doses or revaccination. In 13 of the 24 studies, patients were stratified according to ALL risk groups, which reflect the intensity of chemotherapy. As expected, greater impairment of vaccine-induced immunity was observed in high-risk patients receiving more intensive treatment. And we found a lack of evidence regarding the influenza and yellow fever vaccines. This review identified an opportunity for future studies to evaluate protection for yellow fever, an important arboviral disease in African and Latin American countries.

The first systematic review addressing this topic was published by van Tilburg et al. in 2006 and included eight studies published since 1980 [36]. The proportion of patients retaining protective antibody levels varied widely, ranging from 17–98% for diphtheria, 27–82% for pertussis, 20–98% for tetanus, 62–100% for poliomyelitis, 35–100% for Hib, 29–92% for mumps, 29–60% for measles, and 72–92% for rubella. Most patients responded adequately to revaccination, suggesting immune recovery after chemotherapy. However, due to substantial heterogeneity among studies and small sample sizes, the author concluded that no definitive revaccination policy could be recommended at that time [36].

More recently, Zeng et al. published a second systematic review with meta-analysis in 2025, evaluating the impact of chemotherapy on immunity to prior vaccinations and responses to revaccination in children with ALL. Among 29 eligible studies, eight were included in the meta-analysis. The authors demonstrated a significant decline in pooled seropositive rates after chemotherapy for hepatitis B, hepatitis A, diphtheria, tetanus, pertussis, measles, mumps, rubella, varicella, and pneumococcal conjugate vaccines (*p* < 0.0001). These findings strongly support revaccination strategies in this vulnerable population [37]. Our review included 24 studies, as we excluded studies involving revaccination to focus on the impact of chemotherapy on baseline vaccine-induced immunity.

Current guidelines reflect both available evidence and persistent uncertainties. The Infectious Diseases Society of America (IDSA) recommends that inactivated vaccines be administered three months after completion of chemotherapy, while live vaccines such as measles, mumps, rubella, and varicella may be considered according to specific recommendations and immune recovery. Inactivated influenza vaccine is indicated for induction or consolidation chemotherapy, and pneumococcal conjugate vaccine should be administrated as soon the cancer is diagnosed. In patients treated with anti–B-cell antibodies, vaccination should be delayed for at least six months [38].

Brazilian guidelines similarly acknowledge the lack of consensus and emphasize that vaccination decisions should be individualized and discussed by multidisciplinary teams, ideally ensuring that immunizations are updated before initiation of immunosuppressive therapy whenever possible [39].

European guidelines published by the European Conference on Infections in Leuka-mia emphasize that more than 80% of children with ALL can be cured, and that vaccination aims to protect these patients from infections in a manner comparable to healthy children. Although live vaccines are contraindicated during intensive chemotherapy and responses to inactivated vaccines may be suboptimal, vaccination remains beneficial by reducing infection risk, disease severity, and hospitalizations. Ultimately, vaccination strategies in children and adolescents with ALL have the potential to prevent severe infections and save lives. The European guideline recommend during induction, only hepatitis B vaccine, during maintenance, influenza and pneumococcal vaccines and after the end of chemotherapy, a booster dose of all vaccines [3].

The Department of Global Pediatric Medicine of the St. Jude Children’s Research Hospital surveyed providers in Latin America and the Caribbean to better understand vaccination practices for children with cancer, based on the concern that most cases of childhood cancer occur in low- and middle-income countries and this children’s population is more vulnerable to infections, including VPDs. The study found that healthcare professionals recognize the need to immunize children with cancer; however, national policies to improve immunization coverage to this population are lacking [7].

Our review had some limitations. First, studies addressing revaccination after chemotherapy for ALL are more numerous in the literature, which made the selection of publications specifically evaluating seroprotection after chemotherapy more challenging. In addition, only a few studies assessed protection against pneumococcal disease, an important cause of infection in this population, and we did not identify any studies evaluating protection against yellow fever and influenza without addressing revaccination. The methodology used to assess vaccine-induced protection was not consistently described across all included publications. Additionally, the long-time span covered by the included studies reflects substantial changes in chemotherapy protocols and vaccination schedules, which may contribute to heterogeneity in immune outcomes. Due to the substantial methodological heterogeneity across the included studies, we considered that performing a meta-analysis was not appropriate.

The main strength of this review was the specific focus on vaccine-induced protection after chemotherapy without revaccination, which allowed the evaluation of susceptibility to vaccine-preventable diseases at the end of antineoplastic treatment. Another strength was the inclusion of studies from countries with different socioeconomic conditions, increasing the external validity and global relevance of the findings.

## 5. Conclusions

This systematic review demonstrates a consistent and clinically relevant loss of vaccine-induced immunity in children and adolescents treated for ALL, as we have hypothesized. These findings strongly support the implementation of structured booster or revaccination strategies after chemotherapy, even in previously fully immunized patients. In settings with limited access to serological testing, universal recommendation of vaccines booster doses may represent the most pragmatic approach to reducing the burden of vaccine-preventable diseases in this vulnerable population.

## Figures and Tables

**Table 2:** Protection for tetanus, measles, and Hib.

Disease/Author	Protection Titer	Protection (%)
Tetanus		
Pearson [31]	NR	40.0
Top [28]	≥0.1 IU/mL	70.0
De La Fuente Garcia [25]	≥0.1 IU/mL	46.0
Onoratelli [24]	NR	53.0
Januszkiewicz-Lewandowska [23]	≥0.1 IU/mL	91.0
Lehrnbecher [20]	≥0.1 IU/mL	66.0
Zengin and Sarper [18]	≥0.1 IU/mL	83.3
Calaminus [16]	≥0.1 IU/mL	52.0
Patel [15]	≥0.1 IU/mL	100.0
Ercan [13]	≥0.1 IU/mL	20.0
Ek [4]	≥0.1 IU/mL	33.0
van der Does-van den Berg [11]	≥0.01 IU/mL	100.0
Measles		
Pearson [31]	NR	70.0
Anafy [33]	NR	39.0
Speckhart [32]	NR	64.0
Toret [29]	NR	76.0
Fouda [27]	>13.5 AU/mL	75.4
De La Fuente Garcia [25]	ISR > 1.1	74.0
Onoratelli [24]	NR	46.0
Viana [21]	>1.10 IU/mL	24.1
Cipolotti R	NR	22.0
Zengin and Sarper [18]	>11 VE	16.7
Kosmidis [17]	NR	86.0
Patel [15]	≥120 mIU/mL	71.0
Volc [14]	NR	65.0
Ercan [13]	≥250 mIU/mL	29.0
Nilsson [12]	>0.2 IU/mL	60.0
Hib		
De La Fuente Garcia [25]	>1.0 µg/mL	20.0
Patel [15]	>1.0 µg/mL	87.0
Lehrnbecher [20]	>1.0 µg/mL	55.0
Zengin and Sarper [18]	>1.0 µg/mL	16.7
Ek [4]	>0.15 mg/mL	100.0

Abbreviations: NR: Not reported; ISR: Index standard ratio; VE: Virotech Unit.

## Data Availability

All data included in this systematic review of published studies are available within the manuscript and Supplementary Materials.

## Supplementary Materials

The following supporting information can be downloaded at: https://www.mdpi.com/article/10.3390/vaccines14050419/s1, Table S1: Search strategy; Table S2: Evaluation of studies using the Critical appraisal checklist developed by The Joanna Briggs Institute.
