# Supplementary material for: Vaccine-Induced Immunity in Children and Adolescents After Chemotherapy for Acute Lymphoblastic Leukemia: A Systematic Review
Source: Vaccines (Basel). 2026 May 7;14(5):419. doi: 10.3390/vaccines14050419 (PMC13211610; doi:10.3390/vaccines14050419)
Supplement: Supplementary file 1 [file vaccines-14-00419-s001.zip › vaccines-4167979-supplementary.pdf]

Supplementary Materials:

Table S1. Search strategy

**Database**	**Search strategy**
**MEDLINE /PubMed**	(Infant, Newborn OR Infants, Newborn OR Newborn Infant OR Newborn Infants OR Newborns OR Newborn OR Neonate OR Neonates OR Child, preschool OR Preschool Child OR Children, Preschool OR Preschool Children OR Child OR Children OR Adolescent OR Adolescents OR Adolescence OR Teens OR Teen OR Teenagers OR Teenager OR Youth OR Youths OR Adolescents, Female OR Adolescent, Female OR Female Adolescent OR Female Adolescents OR Adolescents, Male OR Adolescent, Male OR Male Adolescent OR Male Adolescents)<br>AND<br>(Hematologic Neoplasms OR Hematologic Malignancy OR Neoplasms, Hematologic OR Hematologic Neoplasm OR Neoplasm, Hematologic OR Hematological Neoplasms OR Hematological Neoplasm OR Neoplasm, Hematological OR Neoplasms, Hematological OR Malignancies, Hematologic OR Malignancy, Hematologic OR Hematologic Malignancies OR Hematological Malignancies OR Hematological Malignancy OR Malignancies, Hematological OR Malignancy, Hematological OR Hematopoietic Neoplasms OR Neoplasms, Hematopoietic OR Hematopoietic Neoplasm OR Neoplasm, Hematopoietic OR Hematopoietic Malignancies OR Hematopoietic Malignancy OR Malignancies, Hematopoietic OR Malignancy OR Hematopoietic)<br>AND<br>(Antineoplastic Agents OR Agents, Antineoplastic OR Antineoplastic Agent OR Agent, Antineoplastic OR Anticancer Agent OR Agent, Anticancer OR Anticancer Agents OR Agents, Anticancer OR Antineoplastic Drugs OR Drugs, Antineoplastic OR Antineoplastic Drug OR Drug, Antineoplastic OR Antineoplastic; Antitumor Drug OR Drug, Antitumor OR Cancer Chemotherapy Agent OR Agent, Cancer Chemotherapy OR Chemotherapy Agent, Cancer OR Antineoplastics OR Antitumor Agents OR Agents, Antitumor OR Antitumor Drugs OR Drugs, Antitumor OR Cancer Chemotherapy Agents OR Agents, Cancer Chemotherapy OR Chemotherapy Agents, Cancer OR Cancer Chemotherapy Drugs OR Chemotherapy Drugs, Cancer OR Drugs, Cancer Chemotherapy OR Chemotherapeutic Anticancer Agents OR Agents, Chemotherapeutic Anticancer OR Chemotherapeutic Anticancer Drug OR Drug, Chemotherapeutic Anticancer OR Cancer Chemotherapy Drug OR Chemotherapy Drug, Cancer OR Drug, Cancer Chemotherapy OR Antitumor Agent OR Agent, Antitumor)<br>AND

	(Vaccination OR Vaccinations OR Immunization, Active OR Active Immunization OR Active Immunizations OR Immunizations, Active OR Antibody Formation OR Formation, Antibody OR Antibody Response OR Antibody Responses OR Response, Antibody OR Responses, Antibody OR Antibody Production OR Production, Antibody OR Diphtheria Toxoid; Toxoid, Diphtheria OR Diphtheria Vaccine OR Vaccine, Diphtheria OR Pertussis Vaccine OR Vaccine, Pertussis OR Tetanus Toxoid OR Toxoid, Tetanus OR Tetanus Vaccine OR Vaccine, Tetanus OR Poliovirus Vaccines OR Vaccines, Poliovirus OR Poliovirus Vaccine OR Vaccine, Poliovirus OR Haemophilus Vaccines OR Vaccines, Haemophilus OR Hemophilus Vaccines OR Vaccines, Hemophilus OR Haemophilus influenzae Vaccines OR Vaccines, Haemophilus influenzae OR Haemophilus Vaccine Vaccine, Haemophilus OR Mumps Vaccine OR Vaccine, Mumps OR Mumps Vaccine, Inactivated OR Vaccine, Inactivated Mumps OR Mumps Virus Vaccine, Inactivated OR Inactivated Mumps Virus Vaccine OR Inactivated Mumps Vaccine OR Mumps Vaccine, Live Attenuated OR Mumps Virus Vaccine, Live Attenuated OR Live Attenuated Mumps Vaccine OR Live Attenuated Mumps Virus Vaccine OR Measles Vaccine OR Vaccine, Measles OR Rubella Vaccine OR Vaccine, Rubella OR Haemophilus influenzae OR Bacterium influenzae OR Mycobacterium influenzae OR Haemophilus meningitidis OR Influenza-bacillus OR Hemophilus influenzae OR Coccobacillus pfeifferi OR Haemophilus influenzae type b OR Hemophilus influenzae type b OR Hib OR Chickenpox Vaccine OR Vaccine, Chickenpox OR Varicella Vaccine OR Vaccine, Varicella OR Oka Varicella Vaccine OR Vaccine, Oka Varicella OR Varicella Vaccine, Oka Varivax OR Yellow Fever Vaccine OR Fever Vaccine, Yellow OR Vaccine, Yellow Fever OR Immunogenicity, Vaccine OR Vaccine Immunogenicity OR Antigenicity, Vaccine OR Vaccine Antigenicity OR Meningococcal Vaccines OR Vaccines, Meningococcal OR Meningococcal Vaccine OR Vaccine, Meningococcal OR Menactra OR Tetravalent Meningococcal Vaccine OR Meningococcal Vaccine, Tetravalent OR Menomune OR Menveo OR Bivalent Meningococcal Vaccine OR Meningococcal Vaccine, Bivalent OR Meningococcal Polysaccharide Vaccine OR Polysaccharide Vaccine, Meningococcal OR Vaccine, Meningococcal Polysaccharide OR Pneumococcal Vaccines OR Vaccines, Pneumococcal OR Pneumococcal Vaccine OR Vaccine, Pneumococcal OR Pneumovax OR Pneumococcal Polysaccharide Vaccine OR Polysaccharide Vaccine, Pneumococcal OR Vaccine, Pneumococcal Polysaccharide OR Pnu-Imune Vaccine OR Pnu Imune Vaccine OR PnuImune Vaccine)<br>AND<br>(Antibodies OR Antibody Formation OR Formation, Antibody OR Antibody Response OR Antibody Responses OR Response, Antibody OR Responses, Antibody OR Antibody Production OR Production, Antibody OR Immunocompetence OR Immunological Competence OR Competence, Immunological OR Competence, Immunologic OR Immunologic Competence)

**Scopus**	{Infant, Newborn} OR {Infants, Newborn} OR {Newborn Infant} OR {Newborn Infants} OR Newborns OR Newborn OR Neonate OR Neonates OR {Child, preschoo} OR {Preschool Child} OR {Children, Preschool} OR {Preschool Children} OR Child OR Children OR Adolescent OR Adolescents OR Adolescence OR Teens OR Teen OR Teenagers OR Teenager OR Youth OR Youths OR {Adolescents, Female} OR {Adolescent, Female} OR {Female Adolescent} OR {Female Adolescents} OR {Adolescents, Male} OR {Adolescent, Male} OR {Male Adolescent} OR {Male Adolescents}<br>AND<br>{Hematologic Neoplasms} OR {Hematologic Malignancy} OR {Neoplasms, Hematologic} OR {Hematologic Neoplasm} OR {Neoplasm, Hematologic} OR {Hematological Neoplasms} OR {Hematological Neoplasm} OR {Neoplasm, Hematological} OR {Neoplasms, Hematological} OR {Malignancies, Hematologic} OR {Malignancy, Hematologic} OR {Hematologic Malignancies} OR {Hematological Malignancies} OR {Hematological Malignancy} OR {Malignancies, Hematological} OR {Malignancy, Hematological} OR {Hematopoietic Neoplasms} OR {Neoplasms, Hematopoietic} OR {Hematopoietic Neoplasm} OR {Neoplasm, Hematopoietic} OR {Hematopoietic Malignancies} OR {Hematopoietic Malignancy} OR {Malignancies, Hematopoietic} OR Malignancy OR Hematopoietic<br>AND<br>{Antineoplastic Agents} OR {Agents, Antineoplastic} OR {Antineoplastic Agent} OR {Agent, Antineoplastic} OR {Anticancer Agent} OR {Agent, Anticancer} OR {Anticancer Agents} OR {Agents, Anticancer} OR {Antineoplastic Drugs} OR {Drugs, Antineoplastic} OR {Antineoplastic Drug} OR {Drug, Antineoplastic} OR {Antineoplastic; Antitumor Drug} OR {Drug, Antitumor} OR {Cancer Chemotherapy Agent} OR {Agent, Cancer Chemotherapy} OR {Chemotherapy Agent, Cancer} OR Antineoplastics OR {Antitumor Agents} OR {Agents, Antitumor} OR {Antitumor Drugs} OR {Drugs, Antitumor} OR {Cancer Chemotherapy Agents} OR {Agents, Cancer Chemotherapy} OR {Chemotherapy Agents, Cancer} OR {Cancer Chemotherapy Drugs} OR {Chemotherapy Drugs, Cancer} OR {Drugs, Cancer Chemotherapy} OR {Chemotherapeutic Anticancer Agents} OR {Agents, Chemotherapeutic Anticancer} OR {Chemotherapeutic Anticancer Drug} OR {Drug, Chemotherapeutic Anticancer} OR {Cancer Chemotherapy Drug} OR {Chemotherapy Drug, Cancer} OR {Drug, Cancer Chemotherapy} OR {Antitumor Agent} OR {Agent, Antitumor}<br>AND<br>Vaccination OR Vaccinations OR {Immunization, Active} OR {Active Immunization} OR {Active Immunizations} OR {Immunizations, Active} OR {Antibody Formation} OR {Formation, Antibody} OR {Antibody Response} OR {Antibody Responses} OR {Response, Antibody} OR {Responses, Antibody} OR {Antibody Production} OR {Production, Antibody} OR {Diphtheria Toxoid} OR {Toxoid, Diphtheria} OR {Diphtheria Vaccine} OR {Vaccine, Diphtheria} OR

	{Pertussis Vaccine} OR {Vaccine, Pertussis} OR {Tetanus Toxoid} OR {Toxoid, Tetanus} OR {Tetanus Vaccine} OR {Vaccine, Tetanus} OR {Poliovirus Vaccines} OR {Vaccines, Poliovirus} OR {Poliovirus Vaccine} OR {Vaccine, Poliovirus} OR {Haemophilus Vaccines} OR {Vaccines, Haemophilus} OR {Hemophilus Vaccines} OR {Vaccines, Hemophilus} OR {Haemophilus influenzae Vaccines} OR {Vaccines, Haemophilus influenzae} OR {Haemophilus Vaccine} OR {Vaccine, Haemophilus} OR {Mumps Vaccine} OR {Vaccine, Mumps} OR {Mumps Vaccine, Inactivated} OR {Vaccine, Inactivated Mumps} OR {Mumps Virus Vaccine, Inactivated} OR {Inactivated Mumps Virus Vaccine} OR {Inactivated Mumps Vaccine} OR {Mumps Vaccine, Live Attenuated} OR {Mumps Virus Vaccine, Live Attenuated} OR {Live Attenuated Mumps Vaccine} OR {Live Attenuated Mumps Virus Vaccine} OR {Measles Vaccine} OR {Vaccine, Measles} OR {Rubella Vaccine} OR {Vaccine, Rubella} OR {Haemophilus influenzae} OR {Bacterium influenzae} OR {Mycobacterium influenzae} OR {Haemophilus meningitidis} OR {Influenza-bacillus} OR {Hemophilus influenzae} OR {Coccobacillus pfeifferi} OR {Haemophilus influenzae type b} OR {Hemophilus influenzae type b} OR Hib OR {Chickenpox Vaccine} OR {Vaccine, Chickenpox} OR {Varicella Vaccine} OR {Vaccine, Varicella} OR {Oka Varicella Vaccine} OR {Vaccine, Oka Varicella} OR {Varicella Vaccine, Oka Varivax} OR {Yellow Fever Vaccine} OR {Fever Vaccine, Yellow} OR {Vaccine, Yellow Fever} OR {Immunogenicity, Vaccine} OR {Vaccine Immunogenicity} OR {Antigenicity, Vaccine} OR {Vaccine Antigenicity} OR {Meningococcal Vaccines} OR {Vaccines, Meningococcal} OR {Meningococcal Vaccine} OR {Vaccine, Meningococcal} OR Menactra OR {Tetravalent Meningococcal Vaccine} OR {Meningococcal Vaccine, Tetravalent} OR Menomune OR Menveo OR {Bivalent Meningococcal Vaccine} OR {Meningococcal Vaccine, Bivalent} OR {Meningococcal Polysaccharide Vaccine} OR {Polysaccharide Vaccine, Meningococcal} OR {Vaccine, Meningococcal Polysaccharide} OR {Pneumococcal Vaccines} OR {Vaccines, Pneumococcal} OR {Pneumococcal Vaccine} OR {Vaccine, Pneumococcal} OR Pneumovax OR {Pneumococcal Polysaccharide Vaccine} OR {Polysaccharide Vaccine, Pneumococcal} OR {Vaccine, Pneumococcal Polysaccharide} OR {Pnu-Imune Vaccine} OR {Pnu Imune Vaccine} OR {PnuImune Vaccine}<br>AND<br>Antibodies OR {Antibody Formation} OR {Formation, Antibody} OR {Antibody Response} OR {Antibody Responses} OR {Response, Antibody} OR {Responses, Antibody} OR {Antibody Production} OR {Production, Antibody} OR {Immunocompetence} OR {Immunological Competence} OR {Competence, Immunological} OR {Competence, Immunologic} OR {Immunologic Competence}
**EMBASE**	"Infant, Newborn" OR "Infants, Newborn" OR "Newborn Infant" OR "Newborn Infants" OR Newborns OR Newborn OR Neonate OR Neonates OR "Child, preschool" OR "Preschool Child" OR "Children, Preschool" OR

	"Preschool Children" OR Child OR Children OR Adolescent OR Adolescents OR Adolescence OR Teens OR Teen OR Teenagers OR Teenager OR Youth OR Youths OR "Adolescents, Female" OR "Adolescent, Female" OR "Female Adolescent" OR "Female Adolescents" OR "Adolescents, Male" OR "Adolescent, Male" OR "Male Adolescent" OR "Male Adolescents"<br>AND<br>"Hematologic Neoplasms" OR "Hematologic Malignancy" OR "Neoplasms, Hematologic" OR "Hematologic Neoplasm" OR "Neoplasm, Hematologic" OR "Hematological Neoplasms" OR "Hematological Neoplasm" OR "Neoplasm, Hematological" OR "Neoplasms, Hematological" OR "Malignancies, Hematologic" OR "Malignancy, Hematologic" OR "Hematologic Malignancies" OR "Hematological Malignancies" OR "Hematological Malignancy" OR "Malignancies, Hematological" OR "Malignancy, Hematological" OR "Hematopoietic Neoplasms" OR "Neoplasms, Hematopoietic" OR "Hematopoietic Neoplasm" OR "Neoplasm, Hematopoietic" OR "Hematopoietic Malignancies" OR "Hematopoietic Malignancy" OR "Malignancies, Hematopoietic" OR Malignancy OR "Hematopoietic"<br>AND<br>"Antineoplastic Agents" OR "Agents, Antineoplastic" OR "Antineoplastic Agent" OR "Agent, Antineoplastic" OR "Anticancer Agent" OR "Agent, Anticancer" OR "Anticancer Agents" OR "Agents, Anticancer" OR "Antineoplastic Drugs" OR "Drugs, Antineoplastic" OR "Antineoplastic Drug" OR "Drug, Antineoplastic" OR "Antineoplastic; Antitumor Drug" OR "Drug, Antitumor" OR "Cancer Chemotherapy Agent" OR "Agent, Cancer Chemotherapy" OR "Chemotherapy Agent, Cancer" OR Antineoplastics OR "Antitumor Agents" OR "Agents, Antitumor" OR "Antitumor Drugs" OR "Drugs, Antitumor" OR "Cancer Chemotherapy Agents" OR "Agents, Cancer Chemotherapy" OR "Chemotherapy Agents, Cancer" OR "Cancer Chemotherapy Drugs" OR "Chemotherapy Drugs, Cancer" OR "Drugs, Cancer Chemotherapy" OR "Chemotherapeutic Anticancer Agents" OR "Agents, Chemotherapeutic Anticancer" OR "Chemotherapeutic Anticancer Drug" OR "Drug, Chemotherapeutic Anticancer" OR "Cancer Chemotherapy Drug" OR "Chemotherapy Drug, Cancer" OR "Drug, Cancer Chemotherapy" OR "Antitumor Agent" OR "Agent, Antitumor"<br>AND<br>Vaccination OR Vaccinations OR "Immunization, Active" OR "Active Immunization" OR "Active Immunizations" OR "Immunizations, Active" OR "Antibody Formation" OR "Formation, Antibody" OR "Antibody Response" OR "Antibody Responses" OR "Response, Antibody" OR "Responses, Antibody" OR "Antibody Production" OR "Production, Antibody" OR "Diphtheria Toxoid" OR "Toxoid, Diphtheria" OR "Diphtheria Vaccine" OR "Vaccine, Diphtheria" OR "Pertussis Vaccine" OR "Vaccine, Pertussis" OR "Tetanus Toxoid" OR "Toxoid, Tetanus" OR "Tetanus Vaccine" OR "Vaccine,

	Tetanus" OR "Poliovirus Vaccines" OR "Vaccines, Poliovirus" OR "Poliovirus Vaccine" OR "Vaccine, Poliovirus" OR "Haemophilus Vaccines" OR "Vaccines, Haemophilus" OR "Hemophilus Vaccines" OR "Vaccines, Hemophilus" OR "Haemophilus influenzae Vaccines" OR "Vaccines, Haemophilus influenzae" OR "Haemophilus Vaccine" OR "Vaccine, Haemophilus" OR "Mumps Vaccine" OR "Vaccine, Mumps" OR "Mumps Vaccine, Inactivated" OR "Vaccine, Inactivated Mumps" OR "Mumps Virus Vaccine, Inactivated" OR "Inactivated Mumps Virus Vaccine" OR "Inactivated Mumps Vaccine" OR "Mumps Vaccine, Live Attenuated" OR "Mumps Virus Vaccine, Live Attenuated" OR "Live Attenuated Mumps Vaccine" OR "Live Attenuated Mumps Virus Vaccine" OR "Measles Vaccine" OR "Vaccine, Measles" OR "Rubella Vaccine" OR "Vaccine, Rubella" OR "Haemophilus influenzae" OR "Bacterium influenzae" OR "Mycobacterium influenzae" OR "Haemophilus meningitidis" OR "Influenza-bacillus" OR "Hemophilus influenzae" OR "Coccobacillus pfeifferi" OR "Haemophilus influenzae type b" OR "Hemophilus influenzae type b" OR Hib OR "Chickenpox Vaccine" OR "Vaccine, Chickenpox" OR "Varicella Vaccine" OR "Vaccine, Varicella" OR "Oka Varicella Vaccine" OR "Vaccine, Oka Varicella" OR "Varicella Vaccine, Oka Varivax" OR "Yellow Fever Vaccine" OR "Fever Vaccine, Yellow" OR "Vaccine, Yellow Fever" OR "Immunogenicity, Vaccine" OR "Vaccine Immunogenicity" OR "Antigenicity, Vaccine" OR "Vaccine Antigenicity" OR "Meningococcal Vaccines" OR "Vaccines, Meningococcal" OR "Meningococcal Vaccine" OR "Vaccine, Meningococcal" OR Menactra OR "Tetravalent Meningococcal Vaccine" OR "Meningococcal Vaccine, Tetravalent" OR Menomune OR Menveo OR "Bivalent Meningococcal Vaccine" OR "Meningococcal Vaccine, Bivalent" OR "Meningococcal Polysaccharide Vaccine" OR "Polysaccharide Vaccine, Meningococcal" OR "Vaccine, Meningococcal Polysaccharide" OR "Pneumococcal Vaccines" OR "Vaccines, Pneumococcal" OR "Pneumococcal Vaccine" OR "Vaccine, Pneumococcal" OR Pneumovax OR "Pneumococcal Polysaccharide Vaccine" OR "Polysaccharide Vaccine, Pneumococcal" OR "Vaccine, Pneumococcal Polysaccharide" OR "Pnu-Imune Vaccine" OR "Pnu Imune Vaccine" OR "PnuImune Vaccine"<br>AND<br>Antibodies OR "Antibody Formation" OR "Formation, Antibody" OR "Antibody Response" OR "Antibody Responses" OR "Response, Antibody" OR "Responses, Antibody" OR "Antibody Production" OR "Production, Antibody" OR "Immunocompetence" OR "Immunological Competence" OR "Competence, Immunological" OR "Competence, Immunologic" OR "Immunologic Competence"
**Cochrane**	((Infant, Newborn OR Infants, Newborn OR Newborn Infant OR Newborn Infants OR Newborns OR Newborn OR Neonate OR Neonates OR Child, preschool OR Preschool Child OR Children, Preschool OR Preschool Children

	OR Child OR Children OR Adolescent OR Adolescents OR Adolescence OR Teens OR Teen OR Teenagers OR Teenager OR Youth OR Youths OR Adolescents, Female OR Adolescent, Female OR Female Adolescent OR Female Adolescents OR Adolescents, Male OR Adolescent, Male OR Male Adolescent OR Male Adolescents)):<br>AND<br>((Hematologic Neoplasms OR Hematologic Malignancy OR Neoplasms, Hematologic OR Hematologic Neoplasm OR Neoplasm, Hematologic OR Hematological Neoplasms OR Hematological Neoplasm OR Neoplasm, Hematological OR Neoplasms, Hematological OR Malignancies, Hematologic OR Malignancy, Hematologic OR Hematologic Malignancies OR Hematological Malignancies OR Hematological Malignancy OR Malignancies, Hematological OR Malignancy, Hematological OR Hematopoietic Neoplasms OR Neoplasms, Hematopoietic OR Hematopoietic Neoplasm OR Neoplasm, Hematopoietic OR Hematopoietic Malignancies OR Hematopoietic Malignancy OR Malignancies, Hematopoietic OR Malignancy OR Hematopoietic)):<br>AND<br>((Antineoplastic Agents OR Agents, Antineoplastic OR Antineoplastic Agent OR Agent, Antineoplastic OR Anticancer Agent OR Agent, Anticancer OR Anticancer Agents OR Agents, Anticancer OR Antineoplastic Drugs OR Drugs, Antineoplastic OR Antineoplastic Drug OR Drug, Antineoplastic OR Antineoplastic; Antitumor Drug OR Drug, Antitumor OR Cancer Chemotherapy Agent OR Agent, Cancer Chemotherapy OR Chemotherapy Agent, Cancer OR Antineoplastics OR Antitumor Agents OR Agents, Antitumor OR Antitumor Drugs OR Drugs, Antitumor OR Cancer Chemotherapy Agents OR Agents, Cancer Chemotherapy OR Chemotherapy Agents, Cancer OR Cancer Chemotherapy Drugs OR Chemotherapy Drugs, Cancer OR Drugs, Cancer Chemotherapy OR Chemotherapeutic Anticancer Agents OR Agents, Chemotherapeutic Anticancer OR Chemotherapeutic Anticancer Drug OR Drug, Chemotherapeutic Anticancer OR Cancer Chemotherapy Drug OR Chemotherapy Drug, Cancer OR Drug, Cancer Chemotherapy OR Antitumor Agent OR Agent, Antitumor)):ti,ab,kw<br>AND<br>((Vaccination OR Vaccinations OR Immunization, Active OR Active Immunization OR Active Immunizations OR Immunizations, Active OR

	Antibody Formation OR Formation, Antibody OR Antibody Response OR Antibody Responses OR Response, Antibody OR Responses, Antibody OR Antibody Production OR Production, Antibody OR Diphtheria Toxoid; Toxoid, Diphtheria OR Diphtheria Vaccine OR Vaccine, Diphtheria OR Pertussis Vaccine OR Vaccine, Pertussis OR Tetanus Toxoid OR Toxoid, Tetanus OR Tetanus Vaccine OR Vaccine, Tetanus OR Poliovirus Vaccines OR Vaccines, Poliovirus OR Poliovirus Vaccine OR Vaccine, Poliovirus OR Haemophilus Vaccines OR Vaccines, Haemophilus OR Hemophilus Vaccines OR Vaccines, Hemophilus OR Haemophilus influenzae Vaccines OR Vaccines, Haemophilus influenzae OR Haemophilus Vaccine Vaccine, Haemophilus OR Mumps Vaccine OR Vaccine, Mumps OR Mumps Vaccine, Inactivated OR Vaccine, Inactivated Mumps OR Mumps Virus Vaccine, Inactivated OR Inactivated Mumps Virus Vaccine OR Inactivated Mumps Vaccine OR Mumps Vaccine, Live Attenuated OR Mumps Virus Vaccine, Live Attenuated OR Live Attenuated Mumps Vaccine OR Live Attenuated Mumps Virus Vaccine OR Measles Vaccine OR Vaccine, Measles OR Rubella Vaccine OR Vaccine, Rubella OR Haemophilus influenzae OR Bacterium influenzae OR Mycobacterium influenzae OR Haemophilus meningitidis OR Influenza-bacillus OR Hemophilus influenzae OR Coccobacillus pfeifferi OR Haemophilus influenzae type b OR Hemophilus influenzae type b OR Hib OR Chickenpox Vaccine OR Vaccine, Chickenpox OR Varicella Vaccine OR Vaccine, Varicella OR Oka Varicella Vaccine OR Vaccine, Oka Varicella OR Varicella Vaccine, Oka Varivax OR Yellow Fever Vaccine OR Fever Vaccine, Yellow OR Vaccine, Yellow Fever OR Immunogenicity, Vaccine OR Vaccine Immunogenicity OR Antigenicity, Vaccine OR Vaccine Antigenicity OR Meningococcal Vaccines OR Vaccines, Meningococcal OR Meningococcal Vaccine OR Vaccine, Meningococcal OR Menactra OR Tetravalent Meningococcal Vaccine OR Meningococcal Vaccine, Tetravalent OR Menomune OR Menveo OR Bivalent Meningococcal Vaccine OR Meningococcal Vaccine, Bivalent OR Meningococcal Polysaccharide Vaccine OR Polysaccharide Vaccine, Meningococcal OR Vaccine, Meningococcal Polysaccharide OR Pneumococcal Vaccines OR Vaccines, Pneumococcal OR Pneumococcal Vaccine OR Vaccine, Pneumococcal OR Pneumovax OR Pneumococcal Polysaccharide Vaccine OR Polysaccharide Vaccine, Pneumococcal OR Vaccine, Pneumococcal Polysaccharide OR Pnu-Imune Vaccine OR Pnu Imune Vaccine OR PnuImune Vaccine)):

	AND<br>((Antibodies OR Antibody Formation OR Formation, Antibody OR Antibody Response OR Antibody Responses OR Response, Antibody OR Responses, Antibody OR Antibody Production OR Production, Antibody OR Immunocompetence OR Immunological Competence OR Competence, Immunological OR Competence, Immunologic OR Immunologic Competence)) :(Word variations have been searched)"

Table S2. Evaluation of studies using the Critical appraisal checklist developed by The Joanna Briggs Institute

TITLE	1. Was the sample frame appropriate to address the target population?	2. Were study participants sampled in an appropriate way?	3. Was the sample size adequate?	4. Were the study subjects and the setting described in detail?	5. Was the data analysis conducted with sufficient coverage of the identified sample?	6. Were valid methods used for the identification of the condition?	7. Was the condition measured in a standard, reliable way for all participants?	8. Was there appropriate statistical analysis?	9. Was the response rate adequate, and if -t, was the low response rate managed appropriately?
Speckhart *2023*	+	+	+	+	+	+	+	+	+
Anafy 2023	+	+	+	+	+	+	+	+	+
Pearson 2023	+	+	+	+	+	!	!	+	+
Dorval 2021	+	+	+	+	+	+	+	+	+

Touret 2021.	+	+	+	+	+	+	+	+	+
Top 2020	+	+	+	+	+	+	+	+	+
Fouda 2018	+	+	+	+	+	+	+	-	+
Keskin Yildirim 2018	+	+	+	+	+	!	+	+	+
De La Fuente Garcia 2017	+	+	+	+	+	+	+	+	+
Onoratelli 2016.	+	+	+	+	+	+	+	+	+
Januszkiewicz-Lewandowska 2015	+	+	+	+	+	+	+	+	+

Patel 2014	+	+	+	+	+	+	+	!	+
Viana 2012	+	+	+	+	+	+	+	+	+
Patel 2007	+	+	+	+	+	+	+	+	+
Zengin and Sarper 2009	+	+	+	+	+	+	+	+	+
Cipolotti. 2009	+	+	-	+	+	+	+	!	+
Kosmidis 2008	+	+	+	+	+	+	+	+	+
Lehrnbecher 2011	+	+	+	+	+	+	+	+	+

Calaminus 2007	+	+	+	+	+	+	+	+	+
Volc 2006	+	+	!	!	+	+	+	+	+
Ercan 2005	+	+	+	+	+	+	+	+	+
EK 2004	+	+	+	+	+	+	+	+	+
Nilsson 2000	+	+	-	+	+	+	+	+	+
van der Does-van den Berg 1981	+	+	+	+	+	+	+	+	+
